# Metagenome Proteins and Database Contamination

**DOI:** 10.1128/mSphere.00854-20

**Published:** 2020-11-04

**Authors:** Irina R. Arkhipova

**Affiliations:** a Josephine Bay Paul Center for Comparative Molecular Biology and Evolution, Marine Biological Laboratory, Woods Hole, Massachusetts, USA

**Keywords:** MAG, RefSeq, binning, classification, metagenomics, taxonomy, transposons

## Abstract

Continued influx of metagenome-derived proteins with misannotated taxonomy into conventional databases, including RefSeq, threatens to eliminate the value of taxonomy identifiers. To prevent this, urgent efforts should be undertaken by submitters of metagenomic data sets as well as by database managers.

## COMMENTARY

Explosive growth of metagenomic sequences in major databases reflects an impressive progress in development of hardware and software for large-scale shotgun metagenomic sequencing, assembly, and annotation. However, size filtration during microbial sample collection does not provide much protection from eukaryotic cells, such as metazoan sperm or fungal spores, and while bioinformatic tools can correctly assign core eukaryotic or prokaryotic genes to a proper domain of life, they fail to guard against less-conserved sequences systematically misrecognized by computational pipelines. This is especially applicable to poorly studied or rapidly evolving “optional” genes and, most prominently, to transposable elements, often showing deviations in GC-content/k-mer composition and read coverage. With the advent of metagenomics, eukaryotic transposons and understudied gene families are being uncontrollably assigned to prokaryotic genomes and are on track to outpace the well-known problem of eukaryotic genome contamination by associated microorganisms (1). In the era of fully automated analyses with little or no manual curation, huge batches of data, such as soil or ocean metagenomes, undergo taxonomic classification relying on loose criteria, i.e., genome binning based on k-mer and read coverage and taxonomy binning based on similarity to reference data sets (e.g., the curated RefSeq database [2]), neither of which can properly identify eukaryotic mobilome components. Such misassigned sequences, which may comprise over one-tenth of a metagenomic data set (3), could soon outnumber microbial contaminants in eukaryotic genomes, which can amount to a small percentage of a whole-genome-sequencing (WGS) data set but are easier to filter out due to higher density of known genes in prokaryotes.

Inclusion of metagenomes in conventional taxonomy-aware databases defies the very purpose of keeping species-specific database records, on which the scientific community has been relying for many years. Most researchers searching GenBank entries tend to trust the description in the nr protein database as definitively excluding environmental (ENV) samples from WGS projects (4) when there is a separate env_nr database and would not begin their analysis by filtering out misassigned entries based on scattered information from inconsistent, often parenthetical notes in the “keyword” or “source” fields, when the “organism” field presents them with the (incorrect) taxonomic assignment; moreover, even these limited indicators are often absent. Table 1 exemplifies the problem, showing sequences from a Tad clade of eukaryotic non-long-terminal-repeat (non-LTR) retrotransposons, which together with adjacent fungal or animal genes were misassigned to various bacterial (BCT) taxa distributed between ENV and BCT subdivisions, many of which were assigned to RefSeq as “true” bacterial genes, further amplifying the errors. Numerous other transposon superfamilies are displaying similar patterns (not shown), and addition of adjacent genes on the same contigs multiplies the number of misassigned proteins entering RefSeq. With an ever-increasing burden on peer reviewers, screening for such false positives before publication is not too high on the journals’ priority list.

**TABLE 1:** Top 50 misassigned bacterial metagenome entries from the nr protein database (accessed 15 October 2020) queried with a fungal non-LTR retrotransposon (MGR583) from *Pyricularia* (*Magnaporthe*) *grisea* (O13348) harboring the RT_nLTR_like (cd01650), R1_I_EN (cd09077), and Rnase_HI_RT_non_LTR (cd09276) conserved domains^*a*^

DB	SD	Accession no.	Description	Taxonomy	PMID or date
gb	BCT	PON18153.1	Hypothetical protein C2W62_09385	“*Candidatus* Entotheonella serta”	29439203
ref	BCT	WP_190751469.1	Endonuclease/exonuclease/phosphatase	Tolypothrix sp. FACHB-123	29112715
gb	ENV	TKW60303.1	Hypothetical protein DI628_08940	Blastochloris viridis	29180750
gb	ENV	PZR61209.1	Hypothetical protein DI537_52575	Pseudomonas stutzeri	29180750
gb	ENV	TMI85391.1	Hypothetical protein E6H10_03430	*Bacteroidetes* bacterium	31110364
gb	ENV	TMC24228.1	Hypothetical protein E6J34_00855	*Chloroflexi* bacterium	31110364
gb	ENV	MAT33547.1	Hypothetical protein	*Ponticaulis* sp.	29337314
gb	ENV	TMI82166.1	Hypothetical protein E6H10_10105	*Bacteroidetes* bacterium	31110364
gb	ENV	MAD87981.1	Hypothetical protein	Deltaproteobacteria bacterium	29337314
ref	BCT	WP_167367247.1	Endonuclease/exonuclease/phosphatase	*Solemya elarraichensis* gill symbiont	29112715
gb	ENV	TMC17713.1	Hypothetical protein E6J34_18185	*Chloroflexi* bacterium	31110364
gb	ENV	TMI79299.1	Hypothetical protein E6H10_15575	*Bacteroidetes* bacterium	31110364
gb	ENV	RZL28817.1	Hypothetical protein EOP64_03250	*Sphingomonas* sp.	30498029
gb	ENV	MAT33548.1	Hypothetical protein	*Ponticaulis* sp.	29337314
gb	ENV	MBM83139.1	Hypothetical protein	*Planctomycetaceae* bacterium	29337314
ref	BCT	WP_034999417.1	Hypothetical protein	Beijerinckia mobilis	29112715
gb	BCT	PON12600.1	Hypothetical protein C2W62_38760	“*Candidatus* Entotheonella serta”	29439203
gb	ENV	RYF04990.1	Reverse transcriptase family protein	*Oxalobacteraceae* bacterium	30498029
emb	BCT	SHE22257.1	Hypothetical protein BBROOKSOX_612	*Bathymodiolus brooksi* thiotrophic gill symbiont	31611646
ref	BCT	WP_139476173.1	Hypothetical protein	*Bathymodiolus brooksi* thiotrophic gill symbiont	29112715
gb	ENV	RUA04271.1	Hypothetical protein DSY43_06760	*Gammaproteobacteria* bacterium	31073213
gb	ENV	RYE04920.1	Hypothetical protein EOP33_08145	*Rickettsiaceae* bacterium	30498029
ref	BCT	WP_078456858.1	Hypothetical protein	*Solemya velum* gill symbiont	29112715
ref	BCT	WP_143556149.1	Hypothetical protein	*Solemya velum* gill symbiont	29112715
gb	ENV	NQZ78185.1	Endonuclease/exonuclease/phosphatase	*Ekhidna* sp.	2 June 2020
gb	ENV	PYK09849.1	Hypothetical protein DME65_11230	*Verrucomicrobia* bacterium	29899444
gb	ENV	PYY19146.1	Hypothetical protein DMG62_24900	*Acidobacteria* bacterium	29899444
ref	BCT	WP_135568158.1	Hypothetical protein, partial	*Solemya elarraichensis* gill symbiont	29112715
gb	ENV	RYE26883.1	Hypothetical protein EOP45_02990	*Sphingobacteriaceae* bacterium	30498029
ref	BCT	WP_190751467.1	Hypothetical protein	*Tolypothrix* sp. FACHB-123	29112715
ref	BCT	WP_180337602.1	RNA-directed DNA polymerase	*Bathymodiolus brooksi* thiotrophic gill symbiont	29112715
ref	BCT	WP_180337587.1	RNA-directed DNA polymerase	*Bathymodiolus brooksi* thiotrophic gill symbiont	29112715
ref	BCT	WP_101731609.1	Endonuclease/exonuclease/phosphatase	Carnobacterium maltaromaticum	29112715
gb	ENV	RPJ78669.1	Hypothetical protein EHM20_03370	*Alphaproteobacteria* bacterium	30086797
ref	BCT	WP_139476202.1	Reverse transcriptase family protein	*Bathymodiolus brooksi* thiotrophic gill symbiont	29112715
gb	ENV	PZO93396.1	Hypothetical protein DI617_08800	Streptococcus pyogenes	29180750
gb	ENV	MBD0342806.1	Reverse transcriptase family protein	*Microcoleus* sp. Co-bin12	14 September 2020
gb	ENV	PYS85964.1	Hypothetical protein DMF62_17490	*Acidobacteria* bacterium	29899444
gb	ENV	NQY31272.1	RNA-directed DNA polymerase	*Flavobacteriaceae* bacterium	2 June 2020
tpg	ENV	HBI40457.1	TPA: hypothetical protein	Tenacibaculum sp.	30148503
gb	ENV	PYS85881.1	Hypothetical protein DMF62_17715	*Acidobacteria* bacterium	29899444
tpg	ENV	HBI40644.1	TPA: hypothetical protein	*Tenacibaculum* sp.	30148503
ref	BCT	WP_185167786.1	Reverse transcriptase family protein	Proteus mirabilis	29112715
ref	BCT	WP_148116844.1	Endonuclease/exonuclease/phosphatase	Anaplasma phagocytophilum	29112715
gb	ENV	TMI86169.1	Hypothetical protein E6H10_00780	*Bacteroidetes* bacterium	31110364
gb	ENV	NEO82313.1	Hypothetical protein	*Moorea* sp. SIO4G3	16 February 2020
gb	ENV	NQY31470.1	Reverse transcriptase family protein	*Flavobacteriaceae* bacterium	2 June 2020
ref	BCT	WP_141663621.1	Reverse transcriptase family protein	Bacterium 2013Ark19i	29112715
ref	BCT	WP_160869998.1	Reverse transcriptase-like protein	*Pantoea* sp. Taur	29112715
ref	BCT	WP_155403559.1	Hypothetical protein, partial	Piscirickettsia salmonis	29112715

a BLASTP search was limited to Bacteria (taxid:2); database, nr. Data represent all nonredundant GenBank coding DNA sequence (CDS) translations plus PDB plus Swiss-Prot plus PIR plus PRF, excluding environmental samples from WGS projects; hits with E values of >1e−30 are shown. DB, database (GenBank, EMBL, RefSeq, or third party); SD, database subdivision (ENV, environmental samples; BCT, bacterial samples); TPA, third-party annotation. All RefSeq entries are linked to reference 2 (PMID 29112715); unpublished entries unlinked to a PMID show the release date.

Database contamination from metagenomes is an acute problem which is poised to become overwhelming. Unless urgent measures are taken, the value of the taxonomy field in gene annotations will eventually be erased by false positives from misassigned metagenomic bins. Many symbiont WGS projects in fact represent host-associated metagenomes (Table 1 lists many examples of host contigs misassigned to symbiont “isolates”), and it would be prudent in these cases to employ taxonomy classifiers which add host genomes, if sequenced, to a reference data set (3). However, this option is rarely applicable to environmental metagenomes, where eukaryotic contigs can be binned into bacterial metagenome-assembled genomes (MAGs) through less-than-certain matches to a taxonomy reference and occasional coincidences in coverage and k-mer composition—features especially applicable to mobile elements, which may dominate the genomes of understudied eukaryotes. Software tools recognizing eukaryotic sequences in metagenomes (5–7) should be upgraded to include recognition of mobilome coding sequences from curated databases such as Repbase (8), and the corresponding modules should be incorporated into prokaryotic genome annotation pipelines (2). However, while metagenome scientists are striving to improve their genomic and taxonomic binning strategies (3, 9, 10), immediate measures should be taken (i) to ensure separation of fragmented metagenomes from conventional databases, preventing misassigned fragments from entering RefSeq, and (ii) to keep the small contigs out of the rigid taxonomy frames by assigning them to “unclassified sequences” in the taxonomy field. Contigs consisting of <10 to 15 kb are most likely to get misassigned, especially if they lack any “marker genes.” These practices should be implemented before metagenome-derived proteins with random taxonomic assignments overrun species-specific databases to such an extent that the taxonomy field becomes useless.
